# Expression and evolutionary patterns of mycobacteriophage D29 and its temperate close relatives

**DOI:** 10.1186/s12866-017-1131-2

**Published:** 2017-12-02

**Authors:** Rebekah M. Dedrick, Travis N. Mavrich, Wei L. Ng, Graham F. Hatfull

**Affiliations:** Department of Biological Sciences, University of Pittsburgh, Pittsburgh, PA 15260 USA

**Keywords:** Bacteriophage evolution, RNAseq, sRNA

## Abstract

**Background:**

Mycobacteriophages are viruses that infect *Mycobacterium* hosts. A large collection of phages known to infect the same bacterial host strain – *Mycobacterium smegmatis* mc^2^155 – exhibit substantial diversity and characteristically mosaic architectures. The well-studied lytic mycobacteriophage D29 appears to be a deletion derivative of a putative temperate parent, although its parent has yet to be identified.

**Results:**

Here we describe three newly-isolated temperate phages – Kerberos, Pomar16 and StarStuff – that are related to D29, and are predicted to be very close relatives of its putative temperate parent, revealing the repressor and additional genes that are lost in D29. Transcriptional profiles show the patterns of both lysogenic and lytic gene expression and identify highly-expressed, abundant, stable, small non-coding transcripts made from the P_left_ early lytic promoter, and which are toxic to *M. smegmatis*.

**Conclusions:**

Comparative genomics of phages D29, Kerberos, Pomar16 and StarStuff provide insights into bacteriophage evolution, and comparative transcriptomics identifies the pattern of lysogenic and lytic expression with unusual features including highly expressed, small, non-coding RNAs.

**Electronic supplementary material:**

The online version of this article (10.1186/s12866-017-1131-2) contains supplementary material, which is available to authorized users.

## Background

Mycobacteriophages – viruses infecting mycobacterial hosts – have been studied since their first isolation in the 1940’s and now represent one of the largest genomically characterized group of phages [1]. Mycobacteriophage D29 was isolated from a soil sample in California, and shown to infect both saprophytic (e.g. *Mycobacterium smegmatis*) and pathogenic (e.g. *Mycobacterium tuberculosis*) strains of mycobacteria [2]. D29 has been studied extensively and shown to have a broad host range that includes *M. avium*, *M. ulcerans*, *M. scrofulaceum*, *M. fortuitum*, and *M. chelonae* [3] and also to adsorb to *Mycobacterium leprae* [4–6]. It has been used to develop mycobacterial transposition assays [7] and shuttle phasmids [8], and is the basis of a diagnostic test for tuberculosis utilizing D29 amplification [9].

D29 was one of the earliest mycobacteriophage genomes to be completely sequenced [10], following L5 [11]. Because D29 is readily discernible from L5 – especially in forming large clear plaques relative to the smaller turbid plaques of L5 and having different cation requirements [12, 13] – the close genomic relationship to L5 was not anticipated [10]. Moreover, the sequencing of over 1300 mycobacteriophage genomes [14, 15] shows them to be highly diverse, forming at least 30 distinct genomic types, currently represented as Clusters A-Z, and six singleton phages (those with no close relatives). Cluster A is by far the largest group with nearly 500 individual members, and can be divided into at least 18 subclusters [15]. Phages L5 and D29 are both members of Subcluster A2.

Genomic comparison of D29 and L5 suggested that D29 is a clear-plaque derivative of an L5-like temperate parent from which approximately 3.6 kbp has been lost, with one deletion end-point located within the repressor gene [10]. There are several other variations between the two genomes, including insertions/deletions, and some highly divergent regions [10]. The deletion is predicted to confer the clear-plaque phenotype and presumably occurred relatively recently, because the integration apparatus is fully functional [16], and D29 can form lysogens if the L5 repressor is provided [10].

L5, D29, and other Cluster A phages have an unusual system of immunity regulation in which the phage repressor (e.g. L5 gp71) binds to an asymmetric operator site overlapping the P_left_ promoter located at the right end of the genome, and to a large number (24 in L5) of related sites located throughout the genome, predominantly within intergenic regions and oriented in one direction relative to transcription [17]. These are referred to as ‘stoperator’ sites because binding of repressor to sites located between a promoter and a reporter results in down-regulation of the reporter [17]. These are well-conserved between L5 and D29 and share the same consensus (5′-GGTGGc/aTGTCAAG), although the stoperator consensus varies among other Cluster A phages [18]. However, the overall transcriptional patterns of L5 and D29 are not well-understood, including how repressor synthesis is regulated [19] and where late transcription initiates.

Here, we compare the genomes of three Subcluster A2 phages that are very close relatives of the putative temperate parent of phage D29. They all contain the region deleted from D29, are temperate, have intact repressor genes, and are homoimmune with L5. They also have similar transcriptional profiles with D29 and L5, with temporal distinction of early and late transcribed genes. Several regions of small non-coding RNAs of unknown function are also expressed.

## Results

### Relationships of phages StarStuff, Pomar16, and Kerberos to phage D29

**Fig. 1:**
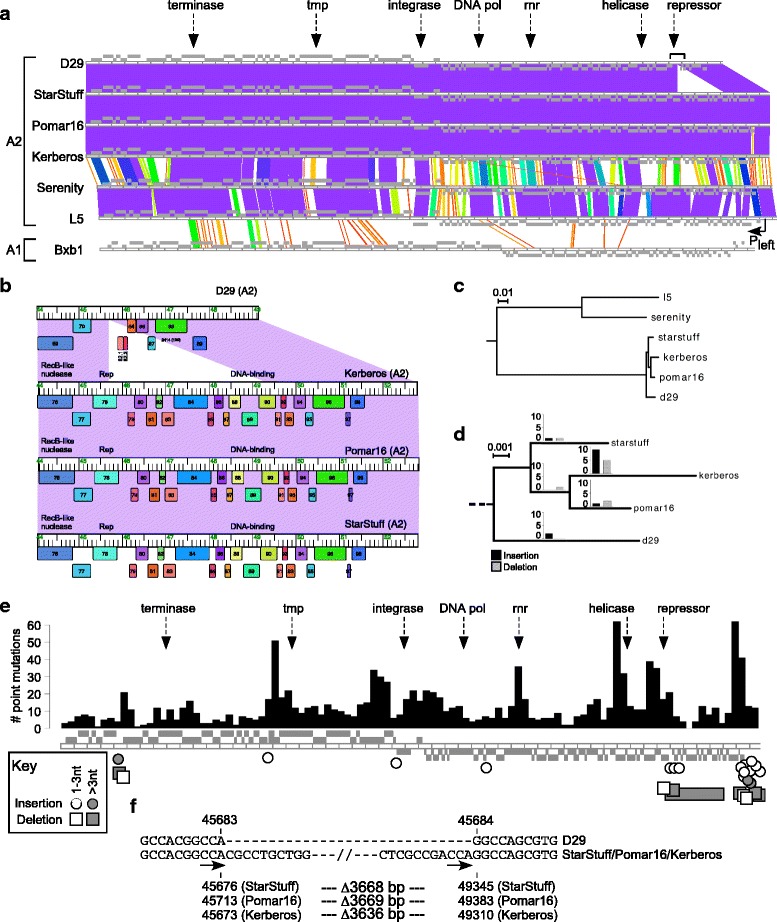
Genomic relationship of D29 to other Cluster A relatives. **a**. Pairwise BLAST-based whole genome alignments of D29 and several Cluster A mycobacteriophages, with the name and subcluster designation indicated. Grey boxes above and below the ruler in each map represent genes transcribed on the top and bottom strands, respectively. The color spectrum between genomes represents pairwise nucleotide similarity, from high similarity (violet) to no detectable similarity (white). Maps were generated using Phamerator (Cresawn et al. 2011). The positions of several genes highly conserved across Cluster A phages are indicated by arrows for reference (tmp = tape measure protein; pol = DNA polymerase; rnr = ribonucleotide reductase). **b**. Expanded view of StarStuff, Pomar16, and Kerberos genes deleted in D29. The predicted functions are shown, but note that the functions of most of the genes in this region are not known. **c-e** Whole genome alignments of the Subcluster A2 phages from panel A were created with Mauve, and mutation events (SNPs and alignment gaps) were identified. **c**. Phylogenetic tree was constructed based on the whole genome alignment. **d**. Alignment gaps were mapped to the phylogenetic branches in the D29 clade (enlarged from panel B) using Count to predict whether gaps were due to insertion or deletion events. Bar charts reflect the total number of predicted events per branch. **e**. Mutation events across the D29 clade are mapped onto the representative StarStuff genome. The histogram reflects the number of SNPs identified. The individual insertion (circle) and deletion (square) events predicted from Count are labeled below and shaded by size. Several highly-conserved genes are labeled as in panel A for reference. **f**. The precise coordinates of the deletion event in D29 that has rendered it lytic (Ford et al. 1998) are labeled in all phages in this clade, with the associated size of the deletion indicated below. Arrows indicate the 3 bp sequence that may have facilitated the deletion

Subcluster A2 includes over 70 individual isolates, and although they are sufficiently similar by average nucleotide identity comparisons to be grouped together, they nonetheless encompass considerable genomic variation [15]. Three of these phages, StarStuff, Pomar16, and Kerberos, are notable in that they show extensive nucleotide sequence similarity across their entire genome spans to phage D29 (Fig. 1a, b). They are not identical, but all were isolated in the same year (2015) and from geographically distinct locations (Pinetown, South Africa; Aibonito, Puerto Rico; and Houston, Texas, respectively, Table 1) [20].

**Table 1:** Characteristics of phages in this paper

Phage	Year Found	Location Found	Genome length (bp)	Cluster, Subcluster	Accession	Reference
L5	1954	Japan	52,297	A, A2	Z18946	Hatfull & Sarkis, 1993
D29	1954	CA, USA	49,127	A, A2	AF022214	Ford et al. 1998
StarStuff	2015	Pinetown, South Africa	52,785	A, A2	KX897981	Jacobs-Sera et al. 2017
Pomar16	2015	Aibonito, Puerto Rico	52,833	A, A2	KX574455	Jacobs-Sera et al. 2017
Kerberos	2015	Houston, TX, USA	52,753	A, A2	KX758538	Jacobs-Sera et al. 2017
Serenity	2012	Pullman, WA, USA	52,088	A, A2	KT381276	Pope et al. 2015

StarStuff, Pomar16, and Kerberos are similarly related to D29. They share 98–99% nucleotide identity with each other and form a monophyletic clade distinct from L5 and its close relative, phage Serenity (Fig. 1c). Within this clade, there are 25 small insertions and deletions (in/dels; Additional file 1: Table S1, Fig. 1d, e), and StarStuff has the fewest in/dels relative to D29 (Fig. 1d). There are also ~500–1000 pairwise single nucleotide polymorphisms (SNPs; Additional file 1: Table S2) at a total of ~1300 positions across the genomes, notably in the tail genes, a segment of the right arms, and the non-coding region at the extreme right ends of the genomes (Fig. 1e; Additional file 2: Figure S1).

Notably, StarStuff, Pomar16, and Kerberos contain intact copies of the repressor gene as well as approximately 3.5 kbp to their right that are absent from D29 (Fig. 1a, b). The segment deleted from D29 is not closely related to other phages, but the closest relative is L5 (80% nucleotide identity spanning 75% of the 3.6 kbp by BlastN). The region is predicted to encode 12 protein coding genes (e.g. StarStuff *79*–*90*, in addition to the repressor), only one of which (StarStuff gp89) has a predicted function (DNA binding protein; Fig. 1b). All of these genes are prominently represented in other Cluster A genomes spanning multiple subclusters, but only two are found in non-Cluster A phages: gene *86* has homologues in Cluster J phages at the extreme right ends of their genomes [21], and gene *90* is related to gene *54* of *Rhodococcus* phage RGL3 [22]. Nucleotide alignments show with high confidence the precise nucleotide positions where the deletion occurred (between D29 coordinates 45,683 and 45,684) resulting in a 3636–3669 bp deletion (Fig. 1f). A simple interpretation is that the deletion resulted from recombination between two directly repeated instances of the 3 bp sequence 5′-CCA. This is an example of the types of recombination events involving little or no homology proposed to contribute to phage genome mosaicism [23].

### StarStuff, L5, and D29 are homoimmune

**Fig. 2:**
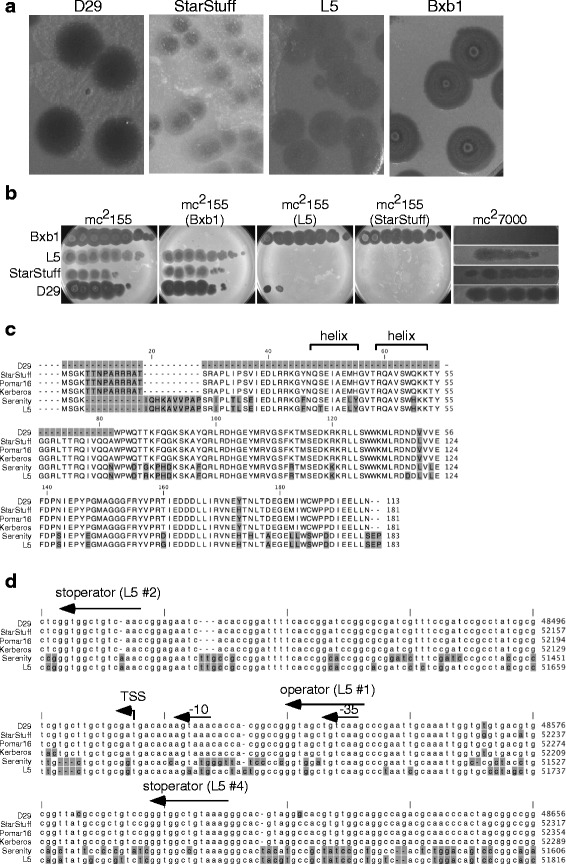
StarStuff lysogen is immune to D29 superinfection. **a**. Comparison of plaques from each phage (D29, StarStuff, L5 and Bxb1) plated on wild type *M. smegmatis* mc^2^155. D29 has large, clear plaques, indicative of its lytic lifestyle; whereas, the temperate phage StarStuff plaques are smaller and more turbid. Both L5 and Bxb1 are also temperate phages. **b**. Each plate represents a lawn of the bacterial strain indicated at the top of each plate. Phages (names on left) were ten-fold serially diluted and spotted onto each lawn. The StarStuff lysogen is immune to infection by D29, due to superinfection immunity. L5, StarStuff and D29 can infect *M. tuberculosis* mc^2^7000. **c**. Amino acid alignment of the immunity repressor from Subcluster A2 phages in Fig. 1a, including the predicted truncated D29 repressor. Alignment positions with variant residues are shaded grey. The predicted helix-turn-helix DNA-binding domain is labeled. Amino acid positions are labeled to the right. The StarStuff, Pomar16 and Kerberos repressors share 78–79% amino acid identity with the L5 repressor. **d**. Whole genome alignment from Fig. 1b-e enlarged to highlight the P_left_ locus at the right end of the genome. Variable nucleotide positions are shaded grey as in panel C. Empirically identified features, such as the operator site, two stoperator sites, and the transcription start site (TSS) are labeled, along with the predicted −10 and −35 promoter elements. Genome coordinates are labeled to the right

StarStuff makes turbid plaques that are somewhat smaller than D29 on *M. smegmatis* (Fig. 2a), and as expected, StarStuff lysogens confer superinfection immunity to itself, D29, and L5, preventing infection at even the highest phage titer (Fig. 2b). We note there is some killing of the L5 lysogen by D29 at the highest titers that is not seen on the StarStuff lysogen (Fig. 2b), although it is unclear if this results from inefficient immunity, lysis from without, or mutants that escape immunity. StarStuff – like D29 and L5 – infects *M. tuberculosis* (Fig. 2b). The StarStuff, Pomar16, and Kerberos repressors are very similar to each other, but share only 78–79% amino acid identity with the L5 repressor (gp71; Fig. 2c); Pomar16 gp78 and Kerberos gp78 are identical to each other and StarStuff gp78 differs by an additional two residues (Fig. 2c).

The repressors of these phages act by binding to an operator site overlapping the P_left_ promoter (Fig. 2d) and approximately 24 stoperator sites positioned throughout the genomes [17]. The stoperators are well conserved between L5 and D29 [17] and also in StarStuff, Pomar16 and Kerberos (Fig. 2d). The operator and 21 stoperator locations are similar in all these phages, StarStuff contains one stoperator within gene *93* that contains a single nucleotide mutation (corresponding to D29 site #34, which is not present in L5, [10]), and Kerberos contains one stoperator with a 1 nt deletion and 2 nt mutations (corresponding to L5/D29 site #6, [10]). Within the region deleted in D29, StarStuff, Pomar16, and Kerberos contain an additional stoperator in the cognate position to L5 site #11 as identified previously [17].

### Transcription profile of L5 lysogenic and lytic growth

**Fig. 3:**
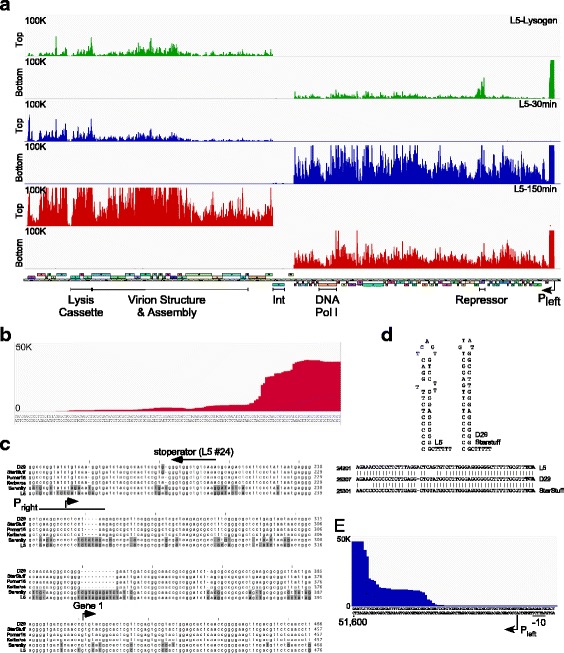
Transcriptomic analysis of L5. **a**. Strand-specific RNAseq analysis of the L5 thermo-inducible lysogen (green), and 30 min (blue) and 150 min (red) after induction; a map of the L5 genome is shown at the bottom. At the left are scale maxima as well as indication of top or bottom strand. **b**. The top strand of the 150 min RNAseq sample indicating the beginning of transcription from P_right_. **c**. Whole genome alignment from Fig. 1b-e enlarged to highlight the P_right_ locus at the left end of the genome, with nucleotide shading as in Fig. 2d. A stoperator site, the beginning of the RNAseq expression data, and the translation start site of gene *1* are labeled. **d**. Putative terminator structures ending rightwards late transcription (e.g. in the L5 *32*–*33* region), and the alignment of the L5, D29 and StarStuff terminator sequences is shown below. **e**. The RNAseq profile at 30 min post induction at the extreme right end of the L5 genome. About 30 bp are under-represented between the beginning of the reads and P_left_, which was mapped previously using S1 nuclease protection and primer extension [19]

**Fig. 4:**
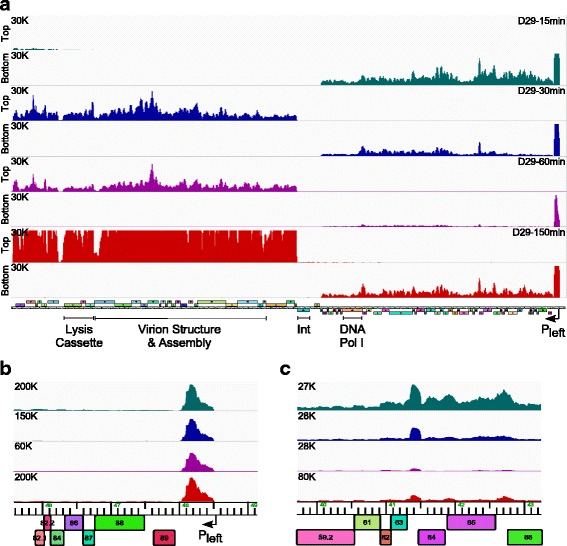
Transcriptomic analysis of D29. **a**. Strand specific RNAseq analysis of D29 (MOI = 3) infected *M. smegmatis* mc^2^155. Time points after adsorption are indicated on the upper right; 15 min (teal), 30 min (blue), 60 min (purple), 150 min (red). At the left are scale maxima and indication of top or bottom strand. The D29 map is shown at the bottom. **b**. Detailed view of the RNAseq reads from the bottom strand at the extreme right end of the D29 genome. Samples are color coordinated with panel A, but note the scale difference from panel **a**. **c**. A detailed view of the RNAseq reads (bottom strand) of the transcribed intergenic region between *63* and *64*

**Fig. 5:**
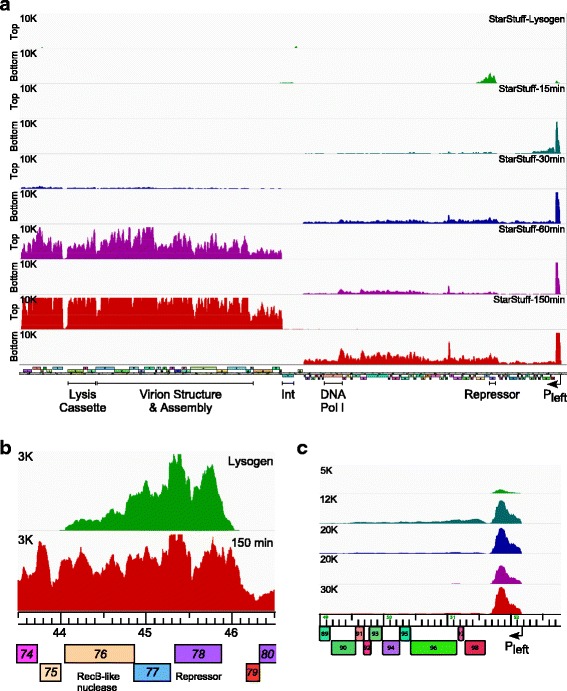
Transcriptomic analysis of StarStuff. **a**. Strand specific RNAseq analysis of a StarStuff lysogen and of StarStuff (MOI = 3) infections of *M. smegmatis* mc^2^155. Time points after adsorption are indicated on the upper right; lysogen (green), 15 min (teal), 30 min (blue), 60 min (purple), 150 min (red). At the left are scale maxima and indication of top or bottom strand. The StarStuff map is shown at the bottom. **b**. A detailed view of the RNAseq reads (bottom strand) of the repressor (*78*), *77* and *76*, a RecB-like nuclease in the lysogen and at 150 min post adsorption. Note the scale difference from panel **a**. **c**. A detailed view of the RNAseq reads from the bottom strand at the extreme right end of the StarStuff genome. Samples are color-coordinated with panel **a**

To further explore the relationships between these Subcluster A2 phages we compared their transcription profiles using strand-specific RNAseq (Figs. 3, 4 and 5). First we used a thermo-inducible mutant of L5 [24] to measure expression during lysogeny, and then at early (30 mins) and late (150 mins) times in lytic growth, which correlate with early and late patterns of phage protein expression [11]. In the lysogen, we see transcripts corresponding to the repressor gene (*71*), although there is considerable expression from all other parts of the genome, reflecting the leakiness of the thermo-inducible repressor allele [24] and a substantial proportion of cells undergoing spontaneous induction even at the permissive temperature (Fig. 3a). At the early time (30 mins) we see strong expression of the leftwards transcribed genes in the right arm (*35*–*89*), and at the late time (150 mins) there is strong expression of the left arm rightwards-transcribed genes (*1*–*32*) including the lysis genes (*10*–*12*) and the virion structure and assembly genes (*13*–*32*) (Fig. 3a). Late transcripts appear to initiate approximately 230 bp in from the left genome end (Fig. 3b, c) and terminate in the gene *32*–*33* intergenic region (Fig. 3d) where a strong transcription terminator was predicted previously [25]. Some late gene transcripts are detected at the 30 min time point, and these are similar in abundance and profile to the uninduced lysogen, and thus likely reflect transcription in the spontaneously induced cells. We observed little or no expression of the integrase gene (*33*) or the adjacent divergently transcribed gene (*34.1*) located at the center of the genome.

An unexpected observation is the apparent extremely high abundance of a ~500 bp region of non–coding DNA at the extreme right end of the genome, located between the P_left_ promoter and gene *89* (Fig. 3a). The number of RNAseq reads in all samples mapping to this locus is greater than those mapping to any other part of the genome, including the capsid gene (*17*). The transcription start site (TSS) of the early lytic promoter P_left_ was previously mapped to coordinate 51,672, by both S1 nuclease protection and primer extension [19], and ~30 bp at the extreme 5′ end is underrepresented in the RNAseq data (Fig. 3e); RNAseq reads start around 51,640, but rise more steeply around coordinate 51,610 (Fig. 3e). Prior analysis showed that the 5′ end of the P_left_ transcript is relatively stable, and readily detectable by S1 nuclease protection 20 min after treatment with rifampicin [19]. The stability of the transcript thus likely contributes to its impressive abundance. It is unclear how the 3′ end is defined and how the transcript levels are reduced by more than 90% for the protein-coding genes downstream, and there is no evident canonical stem-loop terminator similar to that for late gene termination (Fig. 3d). We note that a high level of a similar transcript was observed in other Cluster A phages including phages Kampy [26], SWU1 [27], and RedRock [28].

### Transcription profile of D29 infection

The transcriptional profile of D29 was examined using liquid infection with a multiplicity of infection (MOI) of three, at 15, 30, 60, and 150 min after phage adsorption (Fig. 4a). The overall profile is similar to that observed for L5 following induction of lytic growth. The right arm genes are expressed well at the 15 min time point starting from near the P_left_ promoter [10], and left arm gene expression has started by 30 min, but is strongest after 150 min. Late transcription initiates approximately 230 bp in from the left genome end and ends in the *32.1*–*33* intergenic region, mirroring the L5 expression pattern (Fig. 3). D29 expresses an abundant RNA from the extreme right end of the genome as seen with L5 (Fig. 4b), and we also note an increase of a transcript corresponding to the *63*–*64* intergenic region, relative to the flanking genes (Fig. 4c). This is thus a candidate for a small non-coding RNA, although no putative function is known. We note that sequence reads for the five tRNAs (genes *7,8,9,9.1, 9.2*) are depleted, but this may be an artifact of using only those reads that map uniquely to the phage genome.

### Transcription profile of StarStuff lysogenic and lytic growth

RNAseq analysis of a StarStuff lysogen shows that the repressor (*78*) is the most highly expressed gene, along with the two genes immediately downstream of it (*76*, *77*) (Fig. 5a, b). Although the specific roles of genes *76* and *77* are not known, gene *76* is highly conserved and present in all of the ~500 members of Cluster A (http://phagesdb.org). It is strongly predicted to be a RecB-like nuclease and a member of the Cas4 superfamily. Cas4 is a 5′ to 3′ DNA exonuclease with an iron-sulfur cluster [29], and critical residues including three C-terminal cysteines are absolutely conserved in the Cluster A phage homologues. StarStuff gene *77* is present in only a subset of Cluster A phages, and there is considerable variation in the gene(s) located between the repressor and the putative nuclease among different Cluster A phages. Because genes *76* and *77* are expressed in a lysogen it is plausible that they play a role in prophage-mediated viral defense [30], and we note that the syntenically positioned lambda genes *rexB* and *rexA* are involved in exclusion [31].

In contrast to the thermo-inducible L5 lysogen (Fig. 3a), the lytic genes in the left and right arms are tightly down-regulated in the StarStuff lysogen, and there is only a small amount of transcript from the extreme right end of the genome. There is also some expression of integrase (*37*), and rightwards transcripts from the *37–38* intergenic region, that likely derive from expression of the host tRNA gene at which the phage is integrated. Finally, there is low level expression of the putative HNH endonuclease (*4*) at the left end of the genome (Fig. 5a).

The StarStuff lytic expression profile was determined using liquid infection with a MOI of three, and isolating RNA at 15, 30, 60, and 150 min after adsorption (Fig. 5). Although the general transcription pattern is similar to D29 and L5, there is an apparent delay in lytic development, with higher levels of early gene expression at 30 min than 15 min after infection, and late gene expression is barely detectable at 30 min (Fig. 5a). Furthermore, the number of sequence reads mapping to the phage genome is substantially fewer than with D29 (e.g. at 150 min, 87% of all reads mapped to D29, but only 24% map to StarStuff, see Table S3). A possible explanation is that under these conditions a substantial proportion of infected cells enter the lysogenic state in which lytic gene expression is switched off. However, this is not easy to discern from the RNAseq profiles. First, there is low integrase (gp37) expression at all time points, although there is some detectable expression 15 min after infection, and this may be sufficient for lysogenic establishment. Second, the repressor (gp78) appears to be expressed at all time points during lytic growth together with the other right arm genes (Fig. 5b), as suggested for L5 previously [19] and confirmed with the RNAseq analysis (Fig. 3). Thus, although the RNAseq profile includes cells entering lysogeny as well as entering lytic growth and the repressor expression at later time points could be occurring only in those cells entering lysogeny, when taken together with the L5 transcription pattern, it seems likely that the repressor is transcribed throughout lytic growth; presumably it is either non-translated or otherwise rendered non-functional. We note that like L5 and D29, StarStuff also has a highly abundant transcript near the right genome end (Fig. 5c), and this is easily the most abundant transcript at the 15-min time point, as well as at later infection times (Fig. 5c). There is also a prominent transcript in the non-coding *68*–*69* intergenic region corresponding to the D29 *63*–*64* intergenic non-coding RNA (Fig. 5a).

### Toxic properties of the non-coding region at the right genome end

**Fig. 6:**
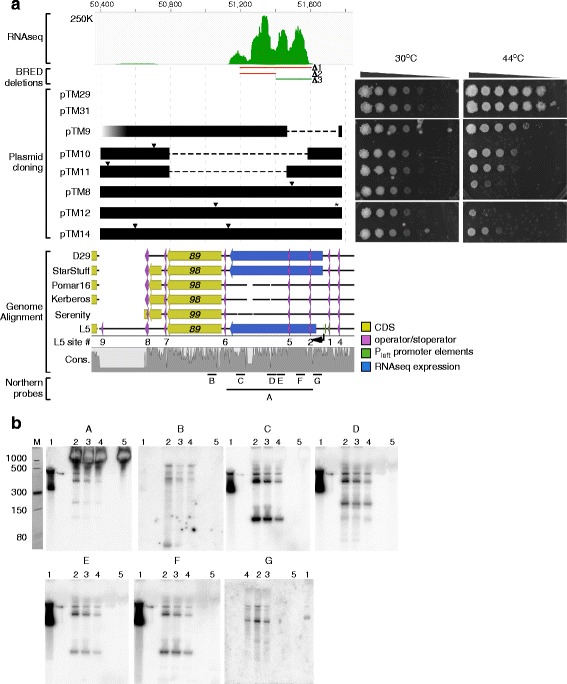
Toxicity of the highly expressed P_left_ locus. **a**. At the top is shown the RNAseq profile of the L5 lysogen at the right end of the genome. Below are indicated the regions targeted for deletion using BRED (Δ1, Δ2, Δ3), only one of which (Δ3, green) could be constructed. Below that are segments of L5 cloned into several plasmids (pTM series), which are aligned with the genomes below. Horizontal dashed lines indicate large deleted segments, triangles indicate single nucleotide mutations, the star indicates a single nucleotide deletion, and the faded region in pTM9 reflects incomplete sequence verification of this locus. Constructs also contain the cloned L5 temperature-sensitive repressor and integration locus, which are not shown. To the right are shown ten-fold serial dilutions of *M. smegmatis* cultures carrying these plasmids, at 30 °C and 44 °C, grown for four days with kanamycin selection. Genome alignments show the positions of predicted genes (yellow arrows), stoperator and operator sites [with corresponding site numbers (Brown et al., 1997)], the L5 P_left_ transcriptional start site (arrow) and promoter elements, and the highly expressed regions from the RNAseq data (blue arrow). Black lines indicate nucleotide sequence with alignment gaps, and the conservation track indicates levels of nucleotide identity across this locus. The size and position of probes used for Northern blots in panel B are labeled below (A-G). **b**. Northern blots were run with a positive control of denatured L5 end dsDNA (Northern probe A) (lane 1), RNA isolated from 30 min post induction (lane 2), 150 min post induction (lane 3), and the uninduced L5 thermo-inducible lysogen (lane 4), as well as an uninfected wildtype mc^2^155 control (lane 5). The probe used for each blot is represented by a letter at the top of each blot and correspond to the lines located at the bottom of the conservation profile which are similarly labeled in panel A. The cropped image of the ethidium bromide stained ssRNA ladder from the denaturing PAGE gel, prior to transfer of the nucleic acids to the membrane, is located at the extreme left of the panel (M)

The highly-transcribed region at the right ends of the genomes is similar in L5, D29 and StarStuff, and we presume that the RNA product acts as a non-coding RNA as there are no open reading frames longer than about 30 codons within the 500 bp transcribed regions, or that are well-conserved among Cluster A phages (see alignment in Additional file 2: Fig. S1). There is considerable sequence divergence within the rightmost 2.1 kb of the genome. This region contains a total of 10 insertions and 5 deletions (60% of which are only 1-2 bp long) and 10% of all SNPs (Fig. 1c-d, Additional file 1: Table S1). Using Bacteriophage Recombineering of Electroporated DNA [BRED [32]], we asked if this ~500 bp (coordinates 51,195–51,619) of L5 is required for lytic growth (Fig. 6a). During the attempted construction of a deletion mutant (Δ1), we identified mixed primary plaques containing both mutant and wild type alleles, but we were unable to subsequently purify the mutant. This behavior is similar to that observed previously for the deletion of genes that are required for lytic growth [32]. Similarly, attempts to construct a smaller deletion (Δ2, coordinates 51,195–51,407) were unsuccessful (Fig. 6a). However, we were able to construct a deletion (Δ3, coordinates 51,407–51,619) removing the right part of the region, showing that at least this segment – and the RNA products produced from it – are not required for lytic growth. We note that there are three major RNA peaks in this 500 bp region (Fig. 6a), and these observations are consistent with the hypothesis that the two rightmost RNA peaks are not required for lytic growth, but the leftmost one is.

To further characterize the region at the right ends of the L5, StarStuff, and D29 genomes we attempted to insert a 1.3 kbp region (coordinates 50,397 to 51,773) from L5 into a plasmid followed by recovery in *E. coli*. We were unable to recover transformants, and we made a second plasmid that also contains the thermo-inducible allele of the L5 repressor gene (*71*) such as to down-regulate P_left_ expression, which might have interfered with clone recovery. Following transformation and recovery of *E. coli* transformants at or below 30 °C, a small number of colonies were obtained, most of which had a noticeable mucoidy appearance. DNA was successfully extracted from these although they gave very poor yield, and the DNA was used to transform *M. smegmatis* and for DNA sequencing.

The region cloned spans coordinates 50,397 to 51,773 and includes the P_left_ promoter, the region that is highly transcribed, gene *89*, and the *88*–*89* intergenic region (Fig. 6a). This also includes the operator at P_left_ (site 1) and seven putative stoperator sites (Fig. 6a) to which the repressor binds [17]. We note that the region between stoperator sites 8 and 9 is deleted in D29, StarStuff, Pomar16, and Kerberos (Fig. 6a) due to an apparent recombination event between the stoperators [10]; in Serenity there is a similar deletion event between sites 8 and 10. None of the clones recovered have the intact wild-type region, and all contain either deletions or point mutations, suggesting that the wild type region is toxic in *E. coli,* even with leaky expression from P_left_. Three of the clones have deletions that have arisen by recombination between stoperator sites (Fig. 6a), but only one removes P_left_, suggesting that the promoter itself is not the reason for the phenotype. The other three clones contain single base substitutions or deletions, which appear to contribute to the partial tolerance of the plasmid in *E. coli* (Fig. 6a).

All of the plasmids could be transformed into *M. smegmatis* and colonies recovered at or below 30 °C. The three deletion clones show relatively mild growth defects at the non-permissive temperature (Fig. 6a, right), with the pTM11 clone showing the greatest defect. The three clones with point mutations show strong growth defects at 44 °C, with pTM12 showing the greatest defect (Fig. 6a, right). This region is thus clearly toxic to the growth of *M. smegmatis*, and the simplest explanation is that this toxicity derives from the strong expression of the stable transcript from P_left_. We cannot rule out that gene *89* contributes to the toxicity, but note that it is only expressed at relatively low levels.

### Non-coding RNAs expressed from genome extreme right ends

The very high abundance of the RNAs expressed from the extreme right ends of the genomes is unusual. To further explore the RNAs expressed, we used a variety of probes to the right end of the L5 genome in Northern blots. First, we created a probe by PCR that spans most of the ~500 bp region from ~60 bp downstream of P_left_ (TSS at 51,672) to near the left end of the transcript peak (Fig. 6b, probe A, coordinates 51,133–51,609). This hybridized to four evident RNA species sized between ~500 nucleotides and ~100 nucleotides (Fig. 6b, probe A), the largest of which corresponds to the peak of reads in the RNAseq data. Next, we used a short probe (Fig. 6b, probe G; 60 nt; coordinates 51,612–51,671) corresponding to the region immediately downstream of P_left_, which hybridized to a similar series of RNA species, confirming the start of transcription at P_left_, and indicating that the smaller species have a common 5′ end and are presumably formed by processing of the larger transcript. A probe from the leftmost region (Fig. 6b, probe B; 60 nt; coordinates 51,023–51,082) that is to the left of the major transcript peak, only weakly hybridizes to larger RNA transcripts that presumably proceed through the signals responsible for forming the 3′ end of the major RNA species. Four smaller probes (~ 60 nt; Fig. 6b, probes C, D, E, F, see Additional file 1: Table S4 for coordinates) all hybridize to a ~500 nucleotide transcript, and several smaller RNAs ranging from 100 to 400 nt.

The simplest explanation for expression of this region is that transcription initiates at P_left_ (TSS at 51,672) and proceeds through to a region (51,140–51,190) where approximately 90% of transcripts terminate. The ~500 bp primary transcript is then at least partially processed into at least four smaller RNA species 100–400 nt long. Transcription of the region occurs early in lytic growth, by 15 mins after D29 infection and 30 min after L5 induction. Substantial transcript is seen in lysogenic L5 growth, which is unexpected because the P_left_ promoter is repressed in lysogeny. It is plausible that it arises from the subset of cells undergoing spontaneous lytic induction and from transiently depressed lysogens, but accumulates because of its considerable stability.

## Discussion

We have described here three mycobacteriophages that are close temperate relatives of the presumed parent of lytic mycobacteriophage D29. It is unclear how or why at least four such closely related phages were isolated in the same year – and not before – all at dispersed geographical locations, but is presumably just by happenstance. However, the ability to isolate and characterize relatively large numbers of phages from a variety of locations is a notable attribute of the SEA-PHAGES program [33], and increases the opportunities for finding such variants. These phages provide insights into the likely structure of the repressor of the direct parent of D29, as well as the genes to its right that are lost in D29.

Mycobacteriophage D29 does not contain a complete repressor gene and does not form lysogens, whereas, StarStuff and L5 are temperate. Using a thermoinducible lysogen, we were able to induce lytic growth of L5 for RNAseq analysis, rather than by infection of D29 or StarStuff. Comparison of the transcription profiles of StarStuff and D29 offers insights into the different life cycles of these phages. In a StarStuff lysogen the most highly expressed genes are the repressor (*78*) and the two downstream genes (*76*–*77*), although some integrase expression is also observed (Fig. 5a, b). Upon infection, transcription initiates from the early lytic promoter, P_left_, resulting in high expression of a stable non-coding RNA. This RNA is the most abundant transcript at all timepoints post-infection (or induction, for L5), suggesting that it is very actively transcribed and perhaps also stably maintained [19].

Lysogenization assays show that L5 can establish lysogeny at greater than 20% efficiency [34]. It is likely that StarStuff forms lysogens as a similar level, and the RNAseq profiles for StarStuff thus likely reflect total expression levels in cells for which there will be different outcomes. It is unclear at what point an irreversible decision is made to complete lytic growth or establish lysogeny, but it is notable that the transcription profile in the StarStuff infection is slower in reaching late lytic expression relative to that in D29. The reason for the delay is unclear, but is unlikely to result from StarStuff genes *79*–*89* that are deleted in D29, as there is only very low expression of these at the earliest time point (15 mins). A further conundrum is that the StarStuff (and L5) repressors appear to be transcribed throughout the growth cycle of infection, as was previously predicted [19]. Although we cannot exclude the possibility that repressor is expressed only in that subset of cells that are going to enter lysogeny and not in lytically growing cells, this seems unlikely, as repressor expression is at a level similar to all of the downstream left arm genes. Presumably, in lytic propagation, either repressor activity is down-regulated at the translational level, or the decision point has passed, such that down-regulation of P_left_ is no longer detrimental to lytic growth.

The abundance of the RNA transcript made from the extreme right ends of the D29, StarStuff and L5 genomes is quite striking. It seems likely that the primary transcript is processed into several smaller RNAs, and the roles for these are not clear, although this region is clearly toxic when expressed in *M. smegmatis* and seemingly also in *E. coli*. This toxicity makes this region difficult to manipulate, because recombinant colonies that are recovered grow extremely slowly and accumulate mutations; attempts to recover additional subclones were unsuccessful, even in *E. coli*. However, similar RNA products were observed previously for the Subcluster A4 phage Kampy [26], Subcluster A2 phage SWU1 [27], and the Subcluster A3 RedRock [28], suggesting this is a common feature for phages with this general genomic architecture (i.e. phages within Cluster A). Because the locus appears to be highly toxic to *M. smegmatis*, it is not expected to be expressed lysogenically, when the repressor is responsible for repression of the P_left_ promoter. In the L5, StarStuff, RedRock, Alma, and Pioneer [28] lysogens, some of this transcript is detected, but it is plausible that it arises from small subsets of cells undergoing spontaneous lytic induction, and accumulated due to its stability. Such highly stable transcripts are somewhat unusual and may be of utility in the design of systems for high levels of recombinant gene expression in mycobacteria.

## Conclusions

Here, we have elucidated the evolutionary relationship of phage D29 to its temperate parents, and determined the patterns of gene expression of D29 and its relatives. Although a role for the highly-expressed, stable, non-coding transcript expressed from the right ends of these genomes during early lytic growth is yet unknown, it is expressed from varied Cluster A mycobacteriophages, suggesting it may play a central role in the lytic propagation of these phages.

## Methods

### Bacterial strains and media

*Mycobacterium smegmatis* mc^2^155 and *Mycobacterium tuberculosis* mc^2^7000 were grown from laboratory stocks as previously described [35–37]. The L5 thermo-inducible lysogen [24] was grown in 200 ml 7H9 media and 1 mM CaCl_2_ (no tween) for ~48 h at 30°C shaking (OD_600_ of 0.7–0.8). Transferred early and late lytic infection cultures to 42°C (shaking) and added pre-warmed media to increase the temperature rapidly.

### Immunity/host range assays

Lysates of each phage were serially diluted in phage buffer and then spotted onto *M. smegmatis* mc^2^155, lysogens, or *M. tuberculosis* mc^2^7000 lawns. Plates were incubated at 37°C. After 24–48 h for *M. smegmatis* or 6 days for *M. tuberculosis*, plates were analyzed for plaque formation.

### Construction of phage mutants

L5 phage mutants were constructed using Bacteriophage Recombineering of Electroporated DNA (BRED) [32] with modifications as in Dedrick et al. 2017.

### RNAseq

Total RNA was isolated from logarithmically growing *M. smegmatis* cells infected with either D29 or StarStuff (MOI of 3) at 15, 30, 60 and 150 min after adsorption or from the L5 thermo-inducible lysogen and induced 30 and 150 min. Removal of DNA was completed using a Turbo-DNase-Free kit (Ambion) according to the manufacturer’s instructions. The depletion of rRNA was completed using the Ribo-Zero Magnetic Kit (Illumina) according to the manufacturer’s instructions. The libraries were constructed using the TruSeq Stranded RNAseq Kit (Illumina) and verified using a BioAnalyzer. The libraries were multiplexed and 4 were run on an Illumina MiSeq for each run. Analysis of the data was as described previously [28].

### Database construction

A Phamerator database of 937 manually annotated actinobacteriophages (Actinobacteriophage_937) was constructed as described previously [38] and is available at http://phamerator.webfactional.com/databases_Hatfull. Amino acid sequences were grouped into gene phamilies (phams) as previously described [14].

### Whole genome alignments

Nucleotide sequences of select genomes were aligned using progressive Mauve [39, 40], implemented through the Mauve graphical user interface (version snapshot_2015–02-25 build 0) with the following settings selected: default seed weight, no seed families, LCBs determined, genomes were assumed to be collinear, with full alignment, with iterative refinement, and sum-of-pairs LCB scoring. Alignment of all six Subcluster A2 genomes was performed for computing phylogenetic whole genome distance in Fig. 1b, P_left_ alignment in Fig. 2d, and P_right_ alignment in Fig. 3c. D29 and its three nearest relatives were aligned separately for identification of single point mutations (‘SNPs’) and alignment gaps that were further analyzed in Count, Excel, and R.

### Phylogenetic tree construction

The phylogenetic tree was constructed on progressive Mauve-aligned whole genome nucleotide sequences using Phyml implemented in Seaview graphical user interface [41] with default settings. Trees were edited using Evolview [42].

### Analyzing insertion and deletion events

Alignment gaps identified by Mauve were manually examined and a sequence gap presence/absence table was constructed. Alignment gaps were mapped to branches in the D29-clade subtree from Fig. 1c in Count [43] using Dollo parsimony. This function, which assumes a single sequence gain event with unlimited sequence deletion events, predicts whether the gap was due to an insertion or deletion event.

### Northern blots

RNA was extracted from the L5 thermo-inducible lysogen and induced 30 min and 150 min after induction as described above. A Low Range ssRNA Ladder (NEB) as well as 10–15 μg total RNA was separated on a 15% TBE-Urea (7 M) polyacrylamide gel at 200 V for 2 h in 1X TBE running buffer (Ambion). The gel was then transferred to a Hybond-N membrane (Amersham Biosciences) by electrophoresis in 0.5X TBE running buffer (Ambion). Hybridization was performed using oligonucleotides that were γ^32^P-ATP labeled with T4 polynucleotide kinase (NEB) for 1 h at 37 °C or – for Northern Probe A - PCR was used to generate the dsDNA product, which was then denatured, and labeled as above. Before adding the probe to the hybridization buffer, it was boiled at 95 °C for 5–10 min, then snap chilled. Blots were pre-hybridized at 37 °C for 30 min and then hybridized with probe for 2 h at 42 °C in Amersham Rapid-hyb buffer (GE Life Sciences) and were washed according to manufacturer’s instructions. The blot was exposed to a phosphorimaging plate overnight, then scanned using a Fuji 5000 Phosphorimager. Blots were stripped with boiling 0.1% SDS and once cooled, equilibrated with DEPC water prior to reprobing.

### Construction of thermo-inducible repressor-controlled P_left_ locus strains

To test the impact of the highly expressed P_left_ locus, plasmid constructs containing this locus under control of the thermo-inducible L5 repressor were constructed. However, creating these constructs was not straightforward, and proceeded in several steps.

First, the thermo-inducible repressor was cloned into the integrating plasmid pMH94 [25], which contains two Sal I restriction sites flanking the L5 integration cassette. The repressor locus was PCR amplified from mc^2^155(L5c^ts43^), a thermo-inducible L5 lysogen [24], at coordinates 44,312–45,192 using Platinum Taq HiFi polymerase and 50 nt primers (oTM91 and oTM92, Additional file 1: Table S4). These primers contained 25 nt at the 5′ end with homology to pMH94 flanking one of the two Sal I cut sites. The 881 nt amplicon was cleaned up using the Nucleospin PCR Cleanup Kit. Gibson assembly was performed according to the manufacturer’s protocol to ligate together the two linearized pMH94 fragments and the repressor amplicon to construct the repressor plasmid (pTM29 and pTM31). Reactions were transformed into *E. coli*, selected with Kanamycin, and verified by restriction digestion, PCR, and sequencing.

Next, the P_left_ locus was cloned into the repressor construct. The P_left_ locus was PCR amplified from the thermo-inducible L5 lysogen strain at coordinates 50,397–51,773 using Platinum Taq HiFi polymerase and 40–41 nt primers (oTM100, oTM101, Additional file 1: Table S4). These primers contain 15–16 nt at the 5′ end that contain Sal I restriction sites. The 1377 nt amplicon was cleaned up using Nucleospin PCR Cleanup Kit, and digested with Sal I. pTM29 was digested at the remaining Sal I restriction site and treated with CIP. The linearized pTM29 and digested amplicon were gel-purified, cleaned up with the Nucleospin Gel Extraction Kit, ligated together, transformed into *E. coli*, and selected with kanamycin. Increased recovery efficiency of transformants occurred at lower growth temperatures of 21^o^ C to 30^o^ C, compared to standard 37 °C. Positive transformants were verified by digestion, PCR, and sequencing, and they exhibit a mucoidy phenotype. No constructs contained the complete wild type P_left_ locus sequence, but instead contained several types of mutations, including single nucleotide mutations, single nucleotide deletions, or large deletions that appear to have been facilitated by the frequent 13 bp stoperator sequences present throughout this locus.

Last, the repressor-only and repressor-controlled P_left_ locus constructs were transformed into electrocompetent *M. smegmatis* mc^2^155 cells and selected with kanamycin. As with the *E. coli* strains, increased recovery efficiency of transformants occurred at lowered growth temperatures of 21^o^ C to 30^o^ C. Positively transformed colonies were noticeably smaller than wild type colonies. Transformants were purified and verified by PCR.

### P_left_ toxicity test

In order to test the impact of the highly expressed locus downstream of P_left_, temperature sensitive growth assays were performed. Liquid cultures of *M. smegmatis* strains were grown from single colonies in 3 ml 7H9 + 4μg/ml kanamycin +0.05% tween 80 at 30^o^ C, shaking at 250 rpm. Cultures were grown for 5 days and diluted to OD_600_ = 0.5. Ten-fold serial dilutions were made for each culture, and 3ul of the 10^0^ to 10^−6^ dilutions were spotted onto two sets of 7H10 + kan plates. One set was incubated at 30^o^ C or 44^o^ C. After four days of growth, images of plates were taken.

## Additional files

Additional file 1: Table S1. Insertions and deletions identified by Mauve whole genome alignment of four D29 relatives, indicating the evolutionary history and nucleotide size of the event. **Table S2.** Total number of single nucleotide polymorphisms (SNPs) for each pairwise comparison identified by Mauve whole genome alignment of four D29 relatives. **Table S3.** A comparison of the stoperators and their coordinates in each genome. **Table S4.** The percentage of RNAseq mapped reads of host vs. phage for the samples indicated on the left. **Table S5.** Oligonucleotides used in this paper to generate PCR fragments, plasmids, as well as Northern blot probes. Coordinates are identified for all Northern blot probes on the right. (DOCX 28 kb)

Additional file 2: Figure S1. Toxic transcript locus alignment. Enhanced view of the 500 bp toxic transcript locus from the whole genome alignment in Fig. 6a. Arrows indicate the beginning and orientation of the strong transcription seen in Figs. 3, 4 and 5. (PDF 430 kb)

## Abbreviations

BRED: Bacteriophage Recombineering of Electroporated DNA
TSS: Transcription start site
