# Supplementary material for: Expression and evolutionary patterns of mycobacteriophage D29 and its temperate close relatives
Source: BMC Microbiol. 2017 Dec 2;17:225. doi: 10.1186/s12866-017-1131-2 (PMC5712189; doi:10.1186/s12866-017-1131-2)
Supplement: Supplementary file 1 — Insertions and deletions identified by Mauve whole genome alignment of four D29 relatives, indicating the evolutionary history and nucleotide size of the event. Table S2. Total number of single nucleotide polymorphisms (SNPs) for each pairwise comparison identified by Mauve whole genome alignment of four D29 relatives. Table S3. A comparison of the stoperators and their coordinates in each genome. Table S4. The percentage of RNAseq mapped reads of host vs. phage for the samples indicated on the left. Table S5. Oligonucleotides used in this paper to generate PCR fragments, plasmids, as well as Northern blot probes. Coordinates are identified for all Northern blot probes on the right. (DOCX 28 kb) [file 12866_2017_1131_MOESM1_ESM.docx]

Expression and evolutionary patterns of mycobacteriophage D29 and its temperate close relatives

Rebekah M. Dedrick^1^, Travis N. Mavrich^1^, Wei L. Ng^1^, and Graham F. Hatfull^1^*

**Supplemental Figures**

Figure S1. Toxic transcript locus alignment. Enhanced view of the 500 bp toxic transcript locus from the whole genome alignment in Fig. 6A. Arrows indicate the beginning and orientation of the strong transcription seen in Figs. 3-5.

**Table S1**: Insertions and deletions identified by Mauve whole genome alignment of four D29 relatives, indicating the evolutionary history and nucleotide size of the event.

|  |  | |  | |  |  | |  |  | |  |
| --- | --- | --- | --- | --- | --- | --- | --- | --- | --- | --- | --- |
| **Insertion/**  **Deletion #** | **Insertion/**  **Deletion** | **Phylogenetic Branch** | | **Size (nt)** | **StarStuff^1^** | **D29^1^** | | **Pomar16^1^** | | **Kerberos^1^** | **Note** |
| 1 | insertion; deletion | Pomar16 | | 47; 5 | 4,399 |  | | 4,399 | |  |  |
| 2 | deletion | Pomar16 | | 1 | 4,498 |  | | 4,540 | |  |  |
| 3 | insertion | Kerberos | | 3 | 15,679 |  | |  | | 15,682 |  |
| 4 | insertion | StarStuff | | 3 | 25,302 |  | |  | |  |  |
| 5 | insertion | D29 | | 1 | 32,068 | 32,074 | |  | |  |  |
| 6 | deletion | Pomar16 | | 1 | 45,317 |  | | 45,355 | |  |  |
| 7 | deletion | D29 | | 3,668 | 45,676 | 45,583 | |  | |  | repressor locus deletion |
| 8 | insertion | StarStuff | | 2 | 45,903 |  | |  | |  | inside D29 deletion |
| 9 | insertion; deletion | Kerberos | | 3; 33 | 46,136 |  | |  | | 46,131 | inside D29 deletion |
| 10 | insertion | Pomar16 | | 3 | 46,670 |  | | 46,705 | |  | inside D29 deletion |
| 11 | insertion | Kerberos | | 3 | 51,288 |  | |  | | 51,253 |  |
| 12 | insertion | Kerberos | | 2 | 51,407 |  | |  | | 51,375 |  |
| 13 | insertion | Kerberos | | 2 | 51,413 |  | |  | | 51,383 |  |
| 14 | insertion | Kerberos | | 1 | 51,418 |  | |  | | 51,390 |  |
| 15 | deletion | Kerberos | | 11 | 51,432 |  | |  | | 51,405 |  |
| 16 | insertion | Kerberos | | 7 | 51,580 |  | |  | | 51,542 |  |
| 17 | insertion | Kerberos | | 5 | 51,588 |  | |  | | 51,557 |  |
| 18 | deletion | Kerberos | | 1 | 51,604 |  | |  | | 51,578 |  |
| 19 | deletion | Kerberos | | 1 | 51,619 |  | |  | | 51,592 |  |
| 20 | insertion | Kerberos | | 1 | 51,629 |  | |  | | 51,601 |  |
| 21 | insertion | ancestor of Kerberos and Pomar16 | | 1 | 52,002 |  | | 52,040 | | 51,975 |  |
| 22 | deletion | StarStuff; Kerberos | | 11 | 52,389 |  | |  | | 52,361 |  |
| 23 | deletion | Kerberos | | 6 | 52,400 |  | |  | | 52,372 |  |
| 24 | insertion | Kerberos | | 2 | 52,539 |  | |  | | 52,505 |  |
| 25 | insertion | D29 | | 1 | 52,680 | 49,030 | |  | |  |  |

^1^Genomic coordinates indicate the aligned nucleotide position adjacent to each event and are provided for the specific genome(s) in which the event occurred, as well as in StarStuff for reference.

**Table S2**: Total number of single nucleotide polymorphisms (SNPs) for each pairwise comparison identified by Mauve whole genome alignment of four D29 relatives.

|  | **Kerberos** | **Pomar16** | **StarStuff** |
| --- | --- | --- | --- |
| **D29** | 950 | 737 | 744 |
| **Kerberos** |  | 587 | 685 |
| **Pomar16** |  |  | 546 |

**Table S3**: A comparison of the stoperators and their coordinates in each genome.

| **D29 site #** |  | **D29** | | **StarStuff** | | **Pomar16** | | **Kerberos** | |  |
| --- | --- | --- | --- | --- | --- | --- | --- | --- | --- | --- |
|  | **Strand** | **Left** | **Right** | **Left** | **Right** | **Left** | **Right** | **Left** | **Right** | **Notes** |
| **1** | bottom | 48536 | 48548 | 52197 | 52209 | 52234 | 52246 | 52169 | 52181 | operator, based on alignment to L5 |
| **2** | bottom | 48423 | 48435 | 52084 | 52096 | 52121 | 52133 | 52056 | 52068 |  |
| **4** | bottom | 48595 | 48607 | 52256 | 52268 | 52293 | 52305 | 52228 | 52240 |  |
| **5** | bottom | 48296 | 48308 | 51957 | 51969 | 51995 | 52007 | 51930 | 51942 |  |
| **6** | bottom | 47954 | 47966 | 51615 | 51627 | 51653 | 51665 | 51587 | 51599 | Kerberos differs from the others |
| **7** | bottom | 47613 | 47625 | 51274 | 51286 | 51312 | 51324 | 51239 | 51251 |  |
| **10** | bottom | 47241 | 47253 | 50902 | 50914 | 50940 | 50952 | 50867 | 50879 |  |
| **12** | bottom | 44042 | 44054 | 44035 | 44047 | 44073 | 44085 | 44032 | 44044 |  |
| **13** | bottom | 43744 | 43756 | 43737 | 43749 | 43775 | 43787 | 43734 | 43746 |  |
| **14** | bottom | 41878 | 41890 | 41871 | 41883 | 41909 | 41921 | 41868 | 41880 |  |
| **15** | bottom | 41326 | 41338 | 41319 | 41331 | 41357 | 41369 | 41316 | 41328 |  |
| **16** | bottom | 39116 | 39128 | 39109 | 39121 | 39147 | 39159 | 39106 | 39118 |  |
| **17** | bottom | 36779 | 36791 | 36772 | 36784 | 36810 | 36822 | 36769 | 36781 |  |
| **18** | bottom | 32881 | 32893 | 32874 | 32886 | 32912 | 32924 | 32871 | 32883 |  |
| **19** | bottom | 29317 | 29329 | 29311 | 29323 | 29349 | 29361 | 29308 | 29320 |  |
| **20** | top | 19026 | 19038 | 19017 | 19029 | 19058 | 19070 | 19017 | 19029 |  |
| **21** | top | 15597 | 15609 | 15588 | 15600 | 15629 | 15641 | 15588 | 15600 |  |
| **22** | top | 13059 | 13071 | 13050 | 13062 | 13091 | 13103 | 13050 | 13062 |  |
| **23** | top | 4680 | 4692 | 4671 | 4683 | 4712 | 4724 | 4671 | 4683 |  |
| **24** | bottom | 199 | 211 | 190 | 202 | 190 | 202 | 190 | 202 |  |
| **31** | bottom | 48759 | 48771 | 52409 | 52421 | 52457 | 52469 | 52375 | 52387 |  |
| **32** | bottom | 47507 | 47519 | 51168 | 51180 | 51206 | 51218 | 51133 | 51145 |  |
| **33** | bottom | 40281 | 40293 | 40274 | 40286 | 40312 | 40324 | 40271 | 40283 |  |
| **34** | bottom | 46294 | 46306 | 49955 | 49967 | 49993 | 50005 | 49920 | 49932 | StarStuff differs from the others |
| **NA** | bottom | NA | NA | 49176 | 49188 | 49214 | 49226 | 49141 | 49153 | site is within D29 deletion locus |

**Table S4**: The percentage of RNAseq mapped reads of host vs. phage for the samples indicated on the left.

|  | **% Reads of Host** | **% Reads of Phage** |
| --- | --- | --- |
| **L5 Lysogen** | 0.84 | 0.14 |
| **L5 30 min** | 0.40 | 0.59 |
| **L5 150 min** | 0.18 | 0.82 |
| **D29 15 min** | 0.85 | 0.15 |
| **D29 30 min** | 0.78 | 0.22 |
| **D29 60 min** | 0.76 | 0.24 |
| **D29 150 min** | 0.13 | 0.87 |
| **StarStuff Lysogen** | 0.99 | 0.00 |
| **StarStuff 15 min** | 0.99 | 0.01 |
| **StarStuff 30 min** | 0.97 | 0.03 |
| **StarStuff 60 min** | 0.89 | 0.11 |
| **StarStuff 150 min** | 0.76 | 0.24 |

**Table S5**: Oligonucleotides used in this paper to generate PCR fragments, plasmids, as well as Northern blot probes. Coordinates are identified for all Northern blot probes on the right.

| **Primer Name** | **Sequence** | **Coordinates** |
| --- | --- | --- |
| oTM91 | CTCGGTACCCGGGGATCCTCTAGAGCAAGCCGGTGTAACGATCTTGAGGC | N/A |
| oTM92 | CAAGCAGAGATGGTGCCCTTGGTGGACACAACCGGTCGTGACTTTTAGGG | N/A |
| oTM100 | CGGATAGCGGGTCGACGTGCCCTTTACAGCCACCGAGAACG | N/A |
| oTM101 | TACCTAGCCTGTCGACGGTGGCTGTCAAGTTGTTGGATAC | N/A |
| L5endFwdpMD70 | CAGCGATCATtccatcgcgcc | N/A |
| L5endRevpMD70 | GGTGAAAATCCGGCGGCAAGA | N/A |
| Northern Probe A Fwd | gtgggtagtcgggaatcgaaccc | L5: 51,133-51,609 |
| Northern Probe A Rev | ggcaagattctccggtttgacagc | L5: 51,133-51,609 |
| Northern Probe B | tgtcttgacgctctcgcgggcagtaacccgaacctcacggtgctcagcgaagactggcat | L5: 51,023-51,082 |
| Northern Probe C | acgttctacggctcagccatagctcagcgatcattccatcgcgccaagagctaccctccc | L5: 51,171-51,230 |
| Northern Probe D | actttaacggcatcgagcttccgccgaccctcagtcctctggcagcgaactaaaggtttg | L5: 51,341-51,400 |
| Northern Probe E | gtcgggctgcggcccttctcggtcttgcgtgattctcactctaccggatgtttcggtggc | L5: 51,402-51,461 |
| Northern Probe F | gcctgatctcaccggtccaagttggtgatgcttgcagcttacccgataaccgggtggctg | L5: 51,530-51,589 |
| Northern Probe G | cggattttcaccggcaccggcacgatcctctcggatccgcctaccgccttgctgctgcgg | L5: 51,612-51,671 |
