# Supplementary figures and images for: Expression and evolutionary patterns of mycobacteriophage D29 and its temperate close relatives
Source: BMC Microbiol. 2017 Dec 2;17:225. doi: 10.1186/s12866-017-1131-2 (PMC5712189; doi:10.1186/s12866-017-1131-2)

47,987

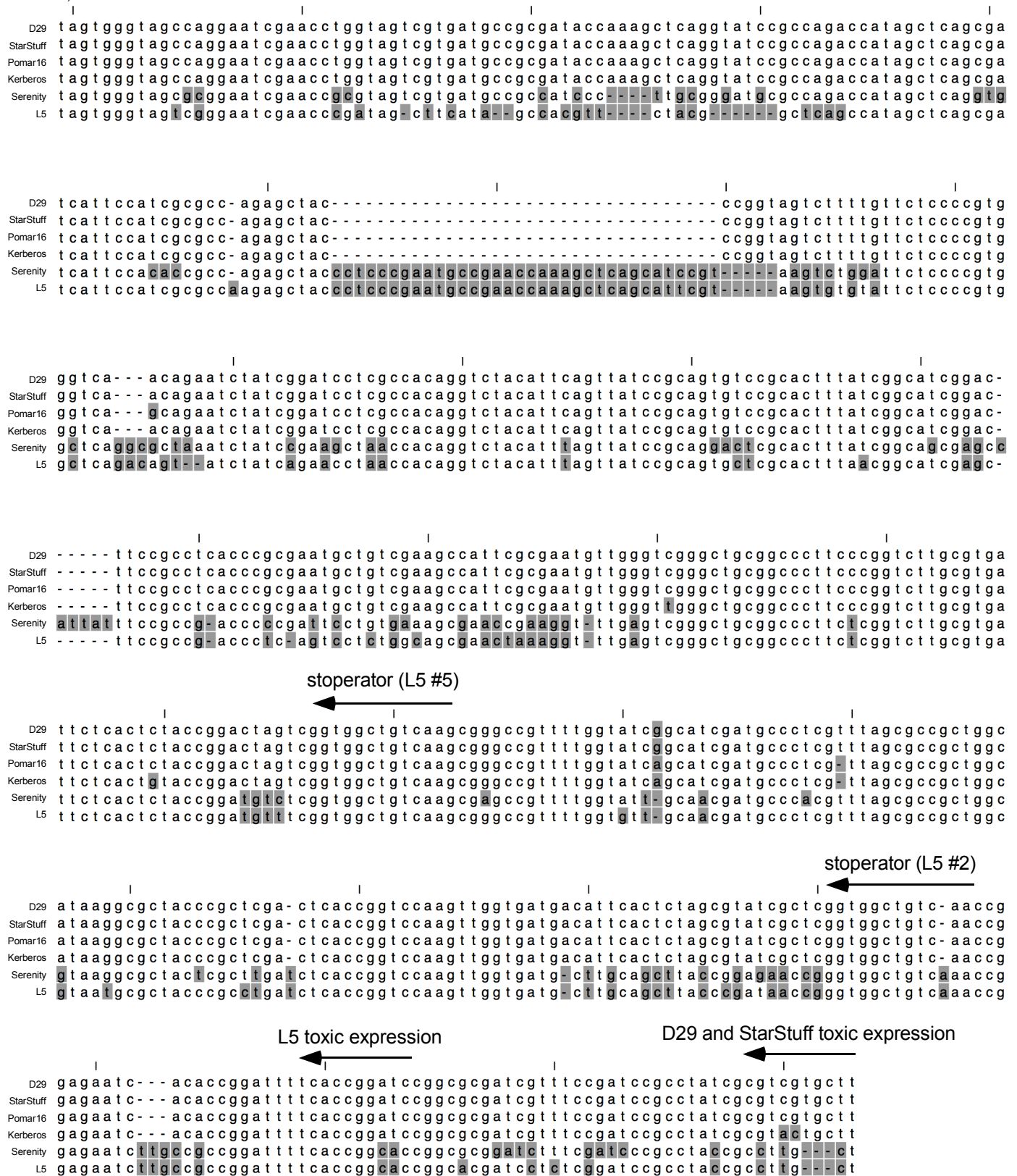

Figure S1

Supplement: Supplementary file 2 — Toxic transcript locus alignment. Enhanced view of the 500 bp toxic transcript locus from the whole genome alignment in Fig. 6a. Arrows indicate the beginning and orientation of the strong transcription seen in Figs. 3, 4 and 5. (PDF 430 kb) [file 12866_2017_1131_MOESM2_ESM.pdf]
